# How should assent to research be sought in low income settings? Perspectives from parents and children in Southern Malawi

**DOI:** 10.1186/s12910-019-0369-7

**Published:** 2019-05-14

**Authors:** Helen Mangochi, Kate Gooding, Aisleen Bennett, Michael Parker, Nicola Desmond, Susan Bull

**Affiliations:** 1 Malawi Liverpool Welcome Trust Clinical Research Programme, P.O. Box 30096, Chichiri, Blantyre, 3 Malawi; 2 Liverpool School of Tropical Medicine, Pembroke Place, Liverpool, L35QA UK; 3 Ethox Centre and Wellcome Centre for Ethics and Humanities, Nuffield Department of Population Health, University of Oxford, Old Road Campus, Oxford, OX37LF UK

**Keywords:** Assent, Consent, Malawi, Paediatric research, Child, *decision making/es [ethics], *Ethics, Research

## Abstract

**Background:**

Paediatric research in low-income countries is essential to tackle high childhood mortality. As with all research, consent is an essential part of ethical practice for paediatric studies. Ethics guidelines recommend that parents or another proxy provide legal consent for children to participate, but that children should be involved in the decision through providing assent. However, there remain uncertainties about how to judge when children are ready to give assent and about appropriate assent processes. Malawi does not yet have detailed guidelines on assent. Understanding perspectives among children and their parents can assist in developing contextually-appropriate assent guidance.

**Methods:**

Qualitative research was conducted with children and parents in three settings in Southern Malawi (low- and high-income urban and rural), to take account of any variations between socioeconomic and cultural contexts. In each setting, interviews were conducted with parents and their children who had participated in paediatric research to understand their experiences of assent and views on appropriate assent practice. Focus groups were also conducted with children and parents, to understand broader social perspectives.

**Results:**

We found widespread support for involving children in decisions on research participation. Participants identified a range of factors that affect children’s capacity to give assent, including intellectual capacity, emotional development, life experience and cultural norms. Age was often mentioned as a consideration, but deemed an unreliable sole indicator of capacity to assent. In relation to appropriate assent processes, participants emphasised considerations such as supporting effective understanding and minimizing harms. Views on how to achieve these aims varied; for example, there were different ideas about the appropriate order in which to approach children and parents, and about whose decision to respect in the event of disagreement.

**Conclusions:**

Parents and children agreed about the value of involving children in decisions on research, and about the need to promote children’s decision-making capacity while respecting parents’ interests in children’s welfare. Developing practical guidance that meets these principles is challenging, particularly given the need for flexible approaches that suit different study types, children’s capacities and family environments. Further discussion within the Malawi research and ethics community will help develop contextually-appropriate guidelines.

## Background

Globally, paediatric health and research development has lagged behind health research in adults [1]. Addressing this research gap and conducting studies with children has the potential to lead to innovations in healthcare which can substantially improve their health and quality of life [2]. There are, however, long-standing and legitimate concerns about including children in research, including worries about the risk of exploitation and imposing burdens and risks on young people [2, 3]. Guidance often recommends that research should only be conducted with children when the findings could not feasibly be obtained through research with adults [3], and there are particular concerns that studies without potential benefit for individual child participants should involve very minimal risk [4]. However, increasingly inclusion of children in research is seen as essential to ensure sufficient evidence for paediatric health interventions. Biological differences between adults and children affect the nature of disease and effects of drugs, so without research, children are at risk of receiving treatments that are ineffective or unsafe [2–4]. Recent international research ethics guidance consequently requires that children and adolescents be included in health-related research, with appropriate safeguards, unless good scientific reasons justify their exclusion [4]. As with all research, informed and voluntary agreement to participate is essential for the ethical conduct of paediatric research. However, when and how children should be involved in these decisions on participation remains an area of debate and uncertainty [4–7].

Many guidelines recommend that a parent or legal proxy should provide authorization for a child to take part in research, and that the agreement (assent) of the child or adolescent should been obtained in an appropriate manner, given their capacity [2]. Definitions of assent vary and are sometimes unclear but assent is often understood as a process of involving children in the decision about research participation [7]. The CIOMS guidelines state that “To give assent means that the child or adolescent is meaningfully engaged in the research discussion in accordance with his or her capacities”, and the Nuffield Council on Bioethics say assent should be “understood as requirements to *involve* children, as much as they wish and are able, in the decision about participation” [2, 4]. While assent can be defined as this process of involvement rather than the resulting decision, in practice it is often equated with agreement to participate [8]. In contrast with consent, however, assent does not have legal force. This relates to an important distinction between legal competence to give consent, and a child’s intellectual and emotional capacity to understand what is involved in research and make an informed decision: the age for legal competence and capacity to make an informed decision often vary [4].

The assent process is intended to have several benefits, including helping to develop a child’s decision-making capacity, providing education, supporting communication between the researcher and the child, and between the child and family members [9]. However, while recognition of the value and importance of assent is increasingly common, there are many uncertainties about how assent should be applied in practice. Areas of ongoing discussion include which children should be asked to assent and how this should be determined, whether some children can provide consent rather than assent, the way in which children should be asked for assent, and appropriate responses to disagreement between children and guardians about research participation [2, 10, 11].

Debates about the assent process and children’s capacity to consent have often involved normative discussion of ethical and legal requirements [12]. Empirical research can inform ethical policy-making by providing a “bottom up” perspective that examines ethical issues as they play out in particular settings [13]. In relation to assent, empirical research suggests that appropriate ways of involving children in decisions on participation are highly context-specific [2]. Key factors that may affect the assent process include relationships between researchers and families, for example, whether the researcher is directly involved in care of the child, the nature of research, including the level of risk, burden and potential benefit for individual children, and the situation of children and their families, for example, gender, health and wider socioeconomic circumstances [2]. Views on assent may also vary with different understandings of childhood between and within countries, including considerable differences in the extent to which children are seen as in need of protection or given responsibilities that might be considered only appropriate for adults in other cultural settings [2]. This influence of context suggests that empirical research to understand views and experiences of assent in specific social, economic and cultural settings is important for informing discussions on assent and development of contextually-appropriate guidelines [2].

To date, however, there has been limited empirical research conducted to inform the design of assent processes in LMICs [10, 14]. Determining appropriate ages and processes for seeking assent can be challenging in such settings. Examples of contextual factors that should be taken into account during the development of assent processes include a lack of formal education and high illiteracy rate (sometimes resulting in children having more education than their parents), a lack of familiarity with medical research, hierarchical social relationships, with children expected to obey their elders, and complex family relationships such as orphanhood, child-headed households and residence with guardians [10]. Widely varying conditions in developmental, social, economic, and cultural situations can dramatically influence children’s physical, social, emotional, and cognitive development [15], affecting appropriate assent and consent processes.

### Context

In Malawi, 53% of the population of 17 million are aged 18 or below. A 2015–2016 health and demographic survey indicated an infant mortality rate of 4.2%, and a 6.3% mortality rate for children aged under five [16]. Research organisations, including the Malawi-Liverpool-Wellcome Trust Clinical Research Programme (MLW), are committed to conducting health research to address high child morbidity and mortality in this setting. During 2015–17, 23 paediatric studies were conducted by MLW alone. Processes for involving children in assent for such research have been informed by national ethics guidance. The Malawi National Health Science Research Committee guidance on conducting research with children states that:

“*Assent to participate in a study must be obtained from minors who are capable of providing assent. In determining whether children are capable of assenting, NHSRC shall take into account the ages, maturity and psychological state of the children involved. However, minors must assent in tandem with parental permission. In certain cases, NHSRC may regard assent by minors to represent an informed consent. Typical case is when such minors are emancipated. These emancipated minors may include those that society may regard them as mature minors; that are legally married; or university students under a defined Malawian adult age of 18 years.*” [17]

When seeking to implement such guidance in practice, numerous questions remain for researchers, for example who should assess the capability of the child to assent, the exact criteria for making such decisions, and how best to seek assent. There is no formal guidance on the minimum age at which assent should be sought in Malawi, and in practice the age varies between studies and is decided in consultation with local ethics committees. These committees often advise researchers to consider seeking assent from children of school going age – typically between the age ranges of 7–17 years; this lower limit has recently changed, and at the time of our data collection, the ethics committee usually advised studies to obtain assent from children age 8 and above. However, there is a lack of empirical research into the views of children and adults in Malawi about whether this age limit is appropriate and about how best to seek assent more generally. Given this uncertainty and lack of evidence, we identified the need for an empirical ethics study to support researchers and ethics committees in implementing the national guidance and to inform development of detailed standard operating procedures about appropriate ways to engage with children in decision-making on research. Empirical research can help researchers and ethics committees to take account of specific contextual factors that affect appropriate processes for assent and consent in this setting, including poverty levels, access to education, a range of cultural views about criteria to define childhood, and differing familial living conditions and life experiences.

## Methods

Qualitative research methods were used to understand the perspectives of children and parents/guardians about appropriate ways of seeking assent to research. Focus group discussions (FGDs) and interviews were conducted to examine views on an appropriate age for assent, the appropriate process, and the relationship between views of assent and typical childhood responsibilities within specific communities.

To take account of any potential variations between different socioeconomic and cultural contexts, data collection took place in three settings: a low-income urban area, a middle-income urban area, and a low-income rural district. In each setting, interviews were conducted with children who had recently taken part in research conducted by MLW and given assent and with their parents or guardians (22 interviews in total, 11 with children and 11 with their parents or guardians). Parents and children were interviewed separately in all but one interview (where the parent wanted to be present during the interview with the child). Children were recruited from three ongoing or recent studies, two observational studies and one clinical trial. The first observational study examined pneumococcal carriage among vaccinated, healthy children aged 5–17 in high, medium and low-income urban settings. The second observational study examined prevalence of lung disease among healthy children aged 5–8 in rural areas who are exposed to smoke from household burning of biomass for their day-to-day cooking needs. The clinical trial included children aged 8–17 who had HIV and some respiratory problems in urban areas; half received a study product while the others received a placebo. The age of our interview participants reflected the age groups included in these studies. For the rural area, we only interviewed children aged 8 years because the study population was restricted to children age 5 to 8 years, and in line with prevailing ethics committee approaches at the time, only those children aged 8 could be asked for assent.

To gather wider views on appropriate assent processes, 10 focus groups were conducted across the different settings with children attending primary and secondary school, and with male and female parents. These parents were not necessarily the parents of the children who took part in focus groups. We did not conduct focus groups with parents in the middle-income setting, partly because fieldwork had to be curtailed due to risks against fieldworkers related to rumours of ‘bloodsucking’ at the time in Malawi [18], and because we felt that no new themes were emerging by that point in data collection.

The sample is indicated in Table 1 below.

**Table 1:** Study participants – sample size, ages and gender

h		Urban middle income setting	Urban low income setting	Rural setting
Focus group discussion (FGD)	Male parents/ guardians	No FGD	1 FGD - 11 participants Age 25–60 years	1 FGD – 9 participants Age 21–42 years
	Female parents/ guardians	No FGD	1 FGD – 8 participants Age 19–48 years	1 FGD – 10 participants Age 20–40 years
	Secondary school children	1 FGD - 8 participants Age 14–17 years 4 male, 4 female	1 FGD - 8 participants Age 14–17 years 3 male, 5 female	1 FGD − 12 participants Age 14–17 years 5 male, 7 female
	Primary School children	1 FGD −12 participants Age 10–14 years 7 male, 5 female	1 FGD − 12 participants Age 10–14 years 6 male, 6 female	1 FGD - 11 participants Age 10–14 years 5 male, 7 female
Interview pairs	Parents/ guardians	3 interviews Age 24–39 years 1 male, 2 female	3 interviews Age 37–50 years 1 male, 2 female	5 interviews Age 34–38 years 2 male, 3 female
	Children	3 interviews Age 10–17 years 3 female	3 interviews Age 13–16 years 2 male, 1 female	5 interviews Age 8 years 2 male, 3 female

As interview participants were enrolled in other MLW studies, they were approached in collaboration with these study teams and invited to take part in our study on assent. Focus group participants were identified and approached through school representatives and community liaison teams, in collaboration with community engagement staff.

**Fig. 1:**
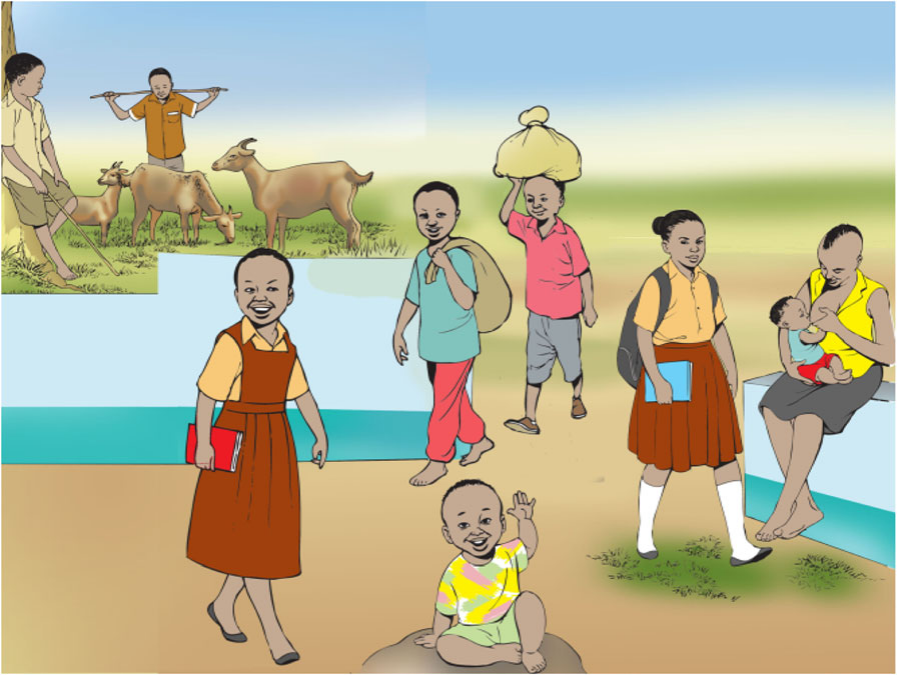
Visual cue to support discussion of contextual understandings of childhood

**Fig. 2:**
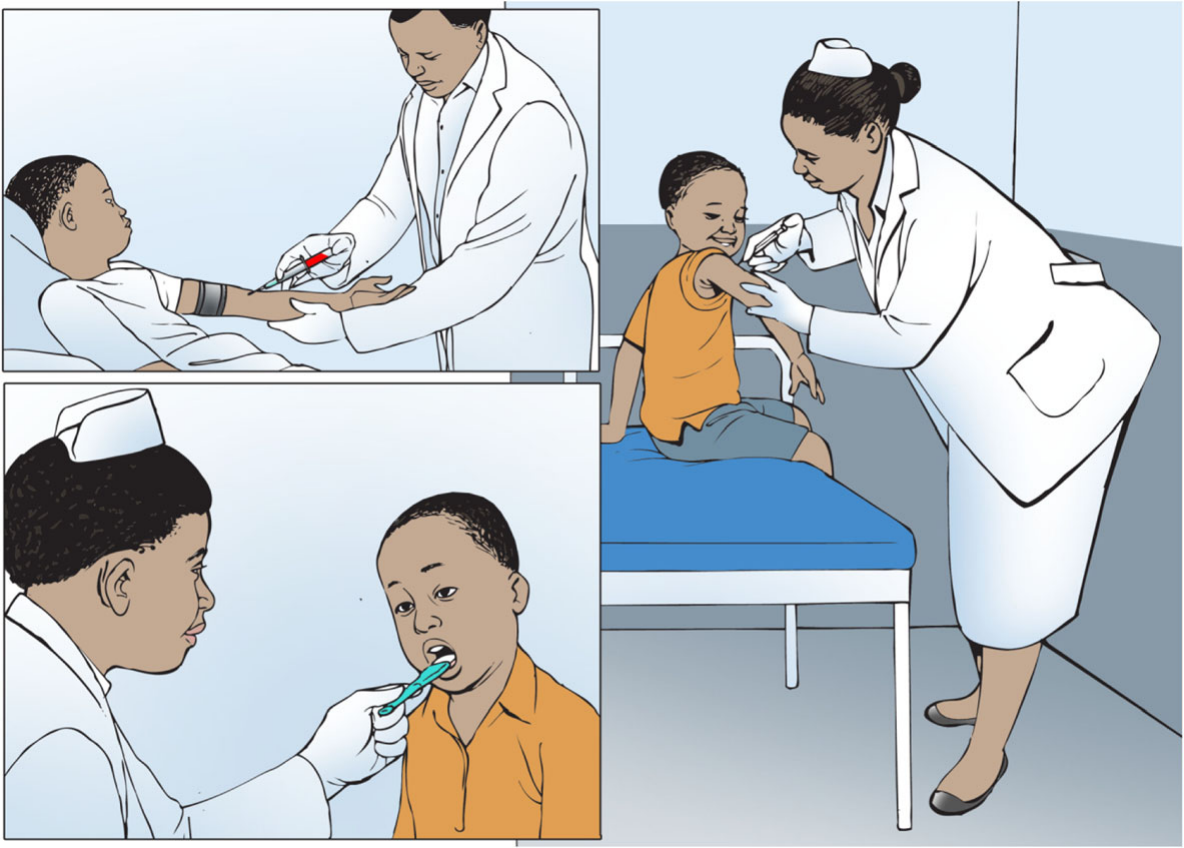
Visual cue used to support discussion of variation in assent between types of research

Interviews lasted approximately 35–40 min, while FDGs ranged from 50 to 70 min. A topic guide was used for interviews and focus groups (see Annex 1). Participants were prompted to discuss who was regarded as a child in their culture and what factors influenced perceived transition to adulthood, including age, daily responsibilities or other criteria. The implications of these views for the process of seeking assent were then explored, asking participants about when children were ready to give assent or consent, and how this process should be conducted, including variation between research designs such as intervention research, observational and qualitative studies. To support discussion, visual cues were used to illustrate varying research designs as well as different stages of childhood and adulthood with attendant responsibilities (see Figs. 1 and 2). These visual cues were developed through collaboration with a local artist. They were introduced by the researcher during interviews to help explain the difference between qualitative studies, observational studies using samples and intervention trials testing new medications. The picture showing children taking different roles was used to discuss who was considered a child in this setting (findings regarding views of childhood will be reported in a separate article).

Data collection was undertaken by a female research nurse with experience of conducting paediatric research and building rapport with parents and children (the lead author). A male fieldworker with experience of working with children in school and community settings assisted with organizing focus groups and taking notes as a backup for audio recordings and to document any additional information on group dynamics that might affect participants’ comments.

Interviews and focus groups were audio recorded, transcribed and translated into English. Transcripts were imported to NVIVO 11 software for data organisation and coding. A sample of transcripts were coded by three researchers, and codes were then discussed to compare interpretations and develop a common coding frame for use with all transcripts. Codes were initially identified primarily inductively, considering issues noticed by researchers as they read through the data. For example, ‘thinking capacity’ and ‘living alone’. Codes were then considered in relation to the research objectives, identifying those that related to questions around when and how children should be included in decisions (for example, grouping codes under larger categories such as ‘when ready to assent’ or ‘order of decision making’). Following coding, framework matrices were used to compare perspectives between individuals and groups. These matrices compared views on issues identified as key themes such as the age at which children could assent or consent, criteria affecting when children are ready to assent, and the appropriate order of assent, to identify any variation in views between parents and children and between different socio-economic settings.

## Results

Study participants had varying ideas about when children are ready to give assent and consent to research participation and about how the assent process should operate. For both when and how children should be involved in decisions on participation, key issues included ability to make informed decisions, protecting children from harm, the rights of children to make independent choices and the roles of parents, and the need to consider the context of individual children and the nature of the research. We first consider views on when children are ready to give assent or consent, and then turn to key considerations for the way children should be involved in decisions on participation and how these decisions should be made.. In the results below we use the term child to refer to participants under the age of 18, but recognize that some of these participants would be viewed as adults by themselves and their families. Views from focus groups and interviews are combined, because opinions shared did not very significantly between the methods.

### When are children ready to give assent or consent?

A core set of considerations underpinning adults’ and children’s views about when children should be asked to provide assent or consent related to children’s maturity and experience, as these affected capacity to understand and decide about participation. Additional important factors affecting views about when children are ready to give assent and consent included ideas related to children’s rights, independence and willingness to listen to parents, the type of research proposed, and the nature of the assent process. These are discussed in turn below.

#### Children’s maturity and experiences

As many research ethics guidelines propose ages at which assent and consent should be sought, participants were prompted to consider relevant age thresholds. Views about the appropriate age for assent and consent varied substantially, and there were different ideas among children, among parents and in each setting, with no consistent differences in suggested age between children and adults or between those in different socioeconomic contexts. Suggested ages at which assent should be sought ranged from 5 to 17 years (with most falling between 8 and 15 years). Similarly, ages at which children could be considered able to consent, rather than assent, to research ranged from 10 years to over 20 years. While some participants suggested that the current minimum age for seeking consent of 18 years was appropriate, many others suggested a lower starting point for consent of somewhere between 12 and 17 years, as outlined below:

*A child who has become a teenager can think properly* … *those at secondary school know what is happening. They know if I do this, it is good and at home people will trust what I have done* [female primary student, low-income urban, FGD]

*I feel that the child should be the one who can reason very well and who is mature. After the age of 16 and 17 going to 18 and above, I feel they can be sensible and understand what they are being told, and make a decision* [male guardian, low-income urban setting, FGD]

While ideas about specific age thresholds for involvement in assent and consent processes varied, there was more consistency in rationales underpinning the suggested ages. Ideas here reflected criteria such as intellectual and decision-making ability, independence, and household circumstances. Several participants emphasized, however, that such criteria are related to, but not wholly determined by, age, making age alone an unreliable indicator of ability to decide on research participation.

*Some are more intelligent than others. That is why you may find that in school, although they may have a similar age and be in the same class, when exams come, some pass and others fail. So although they may have similar ages, the intellect may not be similar.* [male parent, middle-income urban setting, interview]

*In children, the ages can be the same but the thinking can be different* [male primary student, low-income urban setting, FGD]

One core set of criteria affecting views about when children are ready to give assent and consent, indicated by both parents and children in all three settings, related to children’s ability to understand information about the study and make a reasoned decision. This was discussed in relation to both assent (first quote) and consent (second quote).

*Suppose it can be from 10 years going up, they can understand what they are being asked* [female parent, rural setting, interview]

*A 15 year old person is mature and they are able to know that this is right and this wrong* [female primary student, middle-income urban setting, FGD]

A key component of children’s understanding in this context was perceived to be the ability to evaluate potential benefits, harms and risks of a course of action (such as research participation), as discussed below in relation to assent.

*Until they are maybe 10–12, they may be unaware of the effects of something. So this one [daughter, age 9], I see her as in-between, she cannot understand everything well enough to make a decision* [male parent, middle-income urban setting, interview]

Experience of making other health-related decisions was also seen as indicating ability to give consent. For example, some parents felt children could make an independent decision about participation in research once they were making decisions on their own about donating blood or health seeking:

*From 12 and above, children are consulted by the blood transfusion people at school. And at school, parents are not there so a child can decide on their own whether to donate blood* [male parent, rural setting, FGD]

*From 10 and above they are mature and they sometimes go by themselves to the hospital and consult the healthworker and receive medication* [female parent, low-income urban setting, FGD]

Additional life experiences and opportunities for independent decision-making that participants viewed as potentially impacting on capacity to consent before the age of 18 included living within child-led households, being married and having children. In such contexts, children routinely made decisions about their daily living which could increase their capacity to evaluate information provided about research.

*Children without parents are independent; their parents died so they can give ideas [about research] because they think of what to do on a daily basis* [male secondary student, middle-income urban setting, FGD]

*If someone is married, they do not need to get consent from their parents - they are parents themselves and can make independent decisions*. [female parent, rural setting, interview]

*Suppose at 15 or 16 a girl like me gets pregnant and is chased from home. Where does she go? She goes to the man responsible for the pregnancy, and it’s not parents who help you in making decisions when you are there; you do it on your own* [female secondary student, middle-income urban setting, FGD]

However, some participants noted that although such children could provide for themselves, adults have a responsibility to provide advice and support.

*They are just supporting themselves because they are forced to stand on their own by the circumstances,*. *They are still children, they need help or guidance of a certain kind from an adult* [male guardian, low-income urban setting, interview]

#### Rights and independence

Alongside views on children’s maturity and ability to make informed decisions, another area seen as affecting when children are ready to give assent or consent related to ideas about rights and autonomy. Some parents and adults felt that children in particular circumstances or of particular ages had a right to make independent decisions. As with maturity and capacity to make informed choices, this was seen as affected by children’s specific circumstances. For example, some participants mentioned rights to decide in relation to children who are themselves parents.

*A 13 year old girl with a child has the right to make a decision.* [male parent, low-income urban setting, FGD]

While some participants mentioned rights in relation to the importance of respecting autonomy, others referred to rights when discussing parents’ ability to control and impose decisions on their children.

Parents gave rationales for the age at which children should make decisions on participation that related to children ignoring their parents’ views, particularly in relation to independent consent.

*Even though parents should have the final say, with democracy and a lot of talk on the radio about human rights you can’t tell an 18 year old to listen to what I am saying. Our Malawian culture entails that a child, even if they are married, still obeys what the parents say. But democracy changed that… So the child who may need the parent’s say [to take part in research] should be at least 12, but not up to 17–18* [male parent, low-income urban setting, FGD]

As well as changing attitudes to rights, this idea that children would not listen to their parents was also related to a sense of disobedience among children more generally, as mentioned below with regard to consent.

*From 12 to 17 years, children can make decisions, because their bodies have changed and they have a lot of desires. For that reason, even if you block the children from making any decision, they will still do it* [male parent, rural setting, FGD]

*Those who are 17 years old are adults and they don’t listen to what their parents tell them [male primary student, rural income setting, FGD]*

*I think 13 and above, because you can tell them not to take part but the child will insist on that and they may consent to participate without the parents’ knowledge* [female parent, rural setting, FGD]

#### Identifying which children have capacity to assent or consent

Given the difficulty of relying on age, parents and children suggested the need to consider children’s capacity to assent or consent on a case-by-case basis.. Parents in all three settings suggested that they know their children well enough to advise whether they have sufficient understanding to make an independent decision on participation, and also thought that researchers could make an individual assessment.

*Among the children in the household, you can tell by living with them which ones are capable. It’s the same as when the coach of a football team looks at the players’ abilities and knows who to play. So when researchers come to the household, you can say “Denis go and talk to the researchers” because as a parent you know your children. And the researcher too should be friendly and make some jokes and based on what they see, choose to talk to Mary, because they can see that she is capable and can understand what you want to discuss* [male parent, low-income urban setting, FGD]

#### Variations with the nature of research

Views on the level of maturity that children require in order to assent or consent to research varied with the type of study. In general, it was felt that children could make decisions on participation at a younger age with qualitative studies than with research seen as higher risk or involving samples, such as intervention or observation studies.

*[with interviews or focus groups] it is important to just approach the child. I don’t think it can be harmful because it’s not something where the child has an illness and the parents have to be informed. It’s not to do with blood collection or something like that. There isn’t any problem with only getting the child’s view* [male secondary student, low-income urban setting, FGD]

As well as considerations around risk, some people suggested that children should be older to consent for studies involving samples because they might be scared about the samples if approached directly by researchers.

*Start from 16 to 17 years…They are mentally mature and they couldn’t be afraid when asked* [female guardian, rural setting, interview]

*20 and above is the age that that someone can decide independently, unlike 19 and below. In the case of blood collection, some people may be scared to give blood or afraid of the needle, whereas a person who is old enough can see that this not something to be afraid of* [female guardian, low-income urban setting, FGD]

#### Provision of clear information

An important consideration affecting whether children are capable of making a decision about participation is the way researchers explain study information. Participants noted the importance of researchers providing information clearly and appropriately for each child’s capacity and experience, in order to support understanding.

*It will depend on the understanding of the child, whether the child can understand very well. If he or she can’t understand, then there is a need to repeat the information, so that they can understand very well. … But their ability to understand also depends on how the questions are asked. If they are asked in a complicated way, it isn’t good and can confuse the child, because their thinking capacity is not mature enough to understand such things* [male parent low-income urban setting, FGD]

*The child is able to understand […*] *as long as everything is explained well* [male parent, middle-income urban setting, interview]

This indicates the need to consider not just the capacity of the individual child to understand information, but the importance of supporting their understanding through appropriate information provision.

### How should assent be sought?

Once views about determining which children should be asked to assent or consent to research had been explored, participants were asked to reflect on how best to seek assent. Key issues here included the order in which guardians and children should be approached about research and the process if parents and children disagreed.

#### The order of approach

Participants’ had different views about whether the child or guardian should be approached first, or whether to approach them simultaneously. Underpinning these views were considerations of the implications of different orders for promoting understanding, promoting independent decision-making and minimizing potential harms.

In relation to promoting understanding, Some child participants felt children should be approached first because they would have a better understanding of research and so be able to explain it to their parents. This was mentioned particularly in the low-income urban setting, perhaps because children in such low-income settings sometimes have more education than their parents, who had fewer opportunities to attend school.

*It’s better to start with the children. These children will communicate with their parents, because if you approach parents first, some don’t understand clearly what it’s all about. When you discuss it with the children, like for example myself, you give me the information to take home and it will be like discussing just like we are chatting* [male secondary student, low-income urban setting, FGD]

In addition, some children were concerned that if parents were approached first, they might distort the information they share with the child to suit the parent’s interests.

*They may tell you that it’s something bad, whereas if the researchers approach you directly, you can then explain it to your parents* [secondary student, low-income urban setting, FGD]

However, others suggested that a joint approach to parents and children together would support understanding because the child and parent would receive the same information.

*I think they should explain it to both of them because the information will simplify their decisions, whereas if you meet them individually, the information will change* [male secondary student, rural setting, FGD]

Views about the appropriate order of approach were also affected by considerations of promoting children’s abilities to make their own, independent decision. Again, there were contrasting views.. Some participants suggested that children should be approached first because the decision on participation should sit primarily with the child, while others thought children could discuss participation with researchers more openly if their parents had already given permission for the researchers to meet the child.

*We should consult the child first, because the child has the right to accept or deny*. [female primary student, rural setting, FGD]

*The very first thing is to meet the parents and explain what the research is about. Then the parents can freely allow the child to speak to the researchers. When the child is freely released by the parents, they will also be free with you* [male parent, low-income urban setting, FGD]

Others felt meeting parents and children together was most likely to allow children to make an independent decision. Some participants, particularly children, felt parents might refuse without even asking their child if approached alone, or thought parents might discourage participation if children were not present to hear from researchers.

*Tell them together, because some parents might think their child should not participate because the child is too childish, when the child is actually happy to take part. If you tell them together, the parents can ask the child whether they are interested.* [male secondary student, rural setting, FGD]

*It’s better to approach both of them, because if you tell my parents while l am not there and they think the study is difficult, they can threaten me and make me refuse. If you tell us together, everyone can make a decision.* [female secondary student, rural setting, FGD]

In contrast, others raised concerns that in circumstances where children are reluctant to voice different views to their parents, a joint approach could constrain children’s choices.

*There are other children who may not be able to speak in the presence of their parents. They should be approached separately.* [female primary student, low-income urban setting, interview]

While some respondents discussed conditions that would allow children to make an independent decision, others emphasized the value of enabling joint discussion and agreement by approaching parents and children together:

*You can be together with the parents and the child and the researcher, and discuss, then you can come up with one decision and conclusion, it’s supposed to be like that* [male secondary student, middle-income urban setting, FGD]

*They will hear the benefits of the study together. After those discussions, the child will give their decision and the parent will also give their decision so both of you will make one decision*. [male parent, rural setting, FGD]

Although many parents and children emphasized supporting a child’s ability to make or contribute to the decision, many also felt that parents should be approached first because of the inherent importance of respecting parental authority, or because parents had more capacity and experience to make an appropriate decision, without needing children’s input. This view that parents should be consulted first was put forward primarily by parents, but also by some children.

*Anything concerning a child you should consult the parent first [female parent, rural setting, FGD]*

*If you want a child to take part in research you should consult the parents first. A parent has the right to give consent or not, based on their understanding of the research [male parent, rural setting, FGD]*

*A parent is the one who makes a good decision for every child, so you should approach the parent first. [Male parent, rural setting, FGD]*

*They should approach the parents because a child can make the wrong decision, but parents are elders, they know the right decisions and they can discuss things very well with the researcher* [male primary student, rural setting, FGD]

The final set of reasons affecting views about the order of approach in assent processes related to burdens and risks in research. Some participants suggested that parents should be approached first because they may have information about their child’s health that would affect the safety of their participation, and because parents are ultimately responsible for managing any problems that may occur during participation.

*It’s good to consult the parents, because you can attract a 10 year old child with a sweet yet the child doesn’t know that they have a certain problem or suffer from a certain disease. As a result, the child can come back from the research with a weak body … So it’s better to consult the parents because children can just agree to give out blood and cause problems to their bodies* [female parent, rural setting, FGD]

*We should consult the parents first because the parents are the ones who are responsible for the child, so anything bad which might happen to the child can cause a problem for the parents* [female primary student, rural setting, FGD]

*They should approach parents … may be the drugs can have some bad effects, so the parent has to know since they are the ones who take care of the child* [female parent, low-income urban setting, FGD]

In some circumstances however, concerns were raised that approaching guardians first could lead to concerns about the privacy of prospective participants. These discussions focused around children who were in households where there were poor relationships and distrust between children and guardians. Here it was suggested that children should be approached separately to avoid step-parents sharing confidential information, such as a child’s HIV status.

*May be they are your step parents, some parents gossip a lot, start spreading rumours. That’s not good, right? So it’s perhaps good to be approached separately* [female primary student, middle-income urban setting, interview]

#### Managing disagreements about participation in research

When considering how decisions should be made if children and parents disagreed about participation, respondents drew on the themes discussed above about promoting understanding and independent decision-making, and minimizing harms. Many felt that if either the child or parent opposed participation, the child should not take part. Several justifications were given for this approach.

Respecting refusals by parents was seen as important to avoid creating conflict within households.

*You should follow the parent’s decision because the child obeys the parents and if the child insists, the parents will be angry and will no longer support the child* [male primary student, middle-income urban setting, FGD]

*They will shout at you, ‘why did you take part in the research without my consent?’* [male primary student, low-income urban, FGD]

*If the parent has refused but the child is willing to participate, the situation can bring conflict within the household. To avoid this, the researchers can just leave the child if the parent has refused* [male guardian, rural setting, interview]

The need to respect parents’ responsibility for their children was considered particularly important with intervention studies seen as involving higher risks, to manage any problems occurring during participation:

*In the case of testing drugs, when the child is given drugs they may react and create some problems in the body. It can be difficult for the parents, and they may say ‘we told you not to participate but you made your own decision’, and blame the researchers* [female parent, rural setting, FGD]

In contrast, a veto for the child was considered important for respecting the child’s autonomy:

*There is no reason to force the child to participate, that is not respecting the child’s rights*. [male parent, rural setting, FGD]

This idea of autonomy and a child’s right to decide was also mentioned by some children and adults as a reason for respecting a child decision to participate, even when the parent was reluctant, suggesting that parental disagreement may not always be a sufficient basis to exclude a child from participating:

*They should consider the views of the child since has the child has the right as well as the ability to participate [female primary student, rural setting, FGD]*

*If the parents are not willing to participate, then they are not thinking properly, because if the child accepts and the parent refuses then that is not good – it’s a violation of the child’s rights* [female parent, rural setting, interview]

The idea that either the parent’s or child’s disagreement should prevent participation depended partly on the decliner having a clear understanding. If either party did not fully understand what the study involved, then there was potentially more justification for either trying to change their opinion through discussion, or for going ahead despite their concerns. For example, when children had higher levels of education and more understanding of research than their parents and guardians, it was suggested that children could choose another person to support their decision-making, and that their interest in participating should be given more weight.

*The child has the right to take another person who understands about research well, so that they can give consent for them to participate* [female parents, low-income urban setting, FGD]

*The parent may find it difficult to understand, so if the child agrees to participate in something then they should not force the child not to take part because the parents don’t understand* [female parents, low-income urban setting, FGD]

If the child lacked understanding or needing more information, some parents felt they should discuss the study further with the child to ensure they understand the benefits.

*Parents should take the child aside and explain and give guidance on the importance of the research study, so the child can agree* [female parents, low-income urban setting, FGD]

## Discussion

International research ethics guidelines increasingly emphasise the importance both of including children in research, and of ensuring that specific protections are in place to safeguard their rights and welfare [4]. There is widespread consensus that it is important to show respect for children by engaging them in discussions about research participation and seeking their views [2]. It is also seen as critical to safeguard their interests and complement their still developing capacity to make decisions by additionally seeking consent from their parents or guardians [4] It is important to consider how international guidelines should inform the design of assent processes in the Malawian context, given the varied capacities young people may possess in low income settings where responsibilities and living conditions may vary from those in middle and upper income contexts [15]. In many low income settings, including Malawi, children may be entrusted with multiple responsibilities within households but may be side-lined in decision making. Many household have a hierarchical approach to decision-making about matters concerning day to day living, potentially including with research participation [10, 14]. There is clearly potential in many settings for such social frameworks for decision-making to conflict with the international consensus that children and young people should be involved in assent processes for research. However, as discussed above, in this setting we found widespread support from both children and parents for involving children in discussions and deliberations around research participation.

### Assenting and consenting

In this setting there was widespread recognition that a range of factors were important in determining when children should be asked to assent or consent to research. These were related to, but not wholly determined by, age, echoing international concerns about the limitations of age-based guidelines for assent processes [19, 20].

Rather than focusing solely on age, participants discussed a range of factors affecting whether children could and should make decisions, including intellectual capacity life experience and cultural norms around rights and independence. Similar factors affecting capacity to consent have been discussed in the international literature [2, 15, 21]. These perspectives suggest that in Malawi as in other settings, although age is an important indicator in determining appropriate processes for engaging with children about participation, this should be complemented by assessments of individual children, where possible [10].

An important question raised by participants was whether, in some circumstances, children as young as 13–15 could be invited to give consent, rather than assent, to research. In the Southern region of Malawi, 20% percent of children under the age of 18 years do not live with a biological parent, but instead often reside in child, female or elderly led households [16]. These children may have inadequate care and shelter, and may have limited access to proper nutrition, education and healthcare. Without a parent or guardian to provide consent, concerns arise that such children will be excluded from relevant studies. Participants discussed the importance of life experiences in this context, noting that children who did not live with a parent may have extensive relevant decision-making experience and in practice could consent to their own research participation. While it was considered important to respect such decision-making expertise, it was also noted that these children had evolving competencies and there was an obligation on adults to provide them with guidance and support.

### Recognizing interests and promoting welfare

When reflecting on how best to seek assent to research, respondents focused on the need to develop processes that promote effective provision of information, maximize understanding and support for decision-making, and minimize potential harms. Varying views were expressed both about how best to realise these aims, and about appropriate responses to children’s decisions about participation. The need to adapt both the content and the process of assent to the child’s capabilities and the study at hand concurred with views expressed internationally [8, 9].

Views on the order in which children and parents or guardians should be approached to discuss potential participation varied, with some favouring children first, others adults first, and others suggesting children and guardians be approached together. The responses suggested that order should vary depending on the capacities of both the child and parent, the type of research and its potential risks, and the household relationships and approaches to decision-making. Determining the appropriate order to approach children and parents or guardians was viewed as particularly critical when children had stigmatised conditions (such as HIV) and were not living with both biological parents.

When reviewing how decisions should be reached about children’s research participation, similarly to parents in higher income settings, participants discussed a range of views about the weight that should be given to parental decisions and to children’s developing capacities in decision-making [22]. Some respondents focused on the importance of respecting the parents’ ongoing legitimate interest in their children’s decisions and noted the importance of involving children in decision-making, while not necessarily letting a child have the final say. Others discussed the value of promoting discussion between parents and children and reaching a joint decision, or about giving a child’s decision equal weight to that of a parent or guardian. A few participants noted that children may be more educated than their parents, and better able to understand the research and its implications. In such circumstances, questions arose about involvement of other trusted adults in decision-making about research, to ensure that children’s understanding and decision-making was promoted, and their welfare safeguarded.

### Ways forward

These findings suggest that amongst our participants, there is general consensus with international guidance and literature about both the importance of assent, and the need for processes that promote children’s understanding and developing decision-making capacity while also promoting parents’ interests in protecting children’s welfare and minimising harms*.* However, view varied about how best to achieve these aims in practice, and participants emphasised that variations among individual children and families and the research design should inform the design of assent processes. These findings point to the importance of flexibility in guidelines on assent; processes for obtaining assent cannot follow a ‘one size fits all’ approach, but should respond to the type of research, the capacity of child, and the family environment.

In our future work in Malawi, we plan further work to develop policies on assent that take account of this heterogeneity, aiming for guidance that informs the practical implementation of research ethics standards. International research ethics guidance now recognises the importance of promoting the interests of vulnerable populations by including them in research, with appropriate safeguards, unless their exclusion can be scientifically justified [4]. In our setting this highlights the importance of addressing complex ethical questions about what safeguards should be provided, and how best to engage with our most vulnerable children about research, including those without parents or guardians seeking to protect their welfare.

A limitation of this study was that although we sought to consider variation in views about different kinds of research, the wide range of potential differences between studies means not all variations were considered. For example, whether children are healthy or experiencing a condition targeted by the research may affect views on the appropriate assent process, and this is an area for further research. In addition, we concentrated on perspectives among parents and children as a first step in understanding views on assent. The views and experiences of research staff, particularly frontline research nurses and field workers, and of ethics committee members, are also important for informing assent guidelines. To take account of their expertise, we plan to undertake consultation about the findings of this study with additional stakeholders, including research staff, ethics governance teams and national ethics committees. Their contributions and the perspectives of parents and children reported in this article will inform development of guidance on assent processes for MLW and potentially other research institutions in Malawi.

## Conclusions

This study provides empirical findings on the views of parents and children regarding appropriate processes for involving children in decisions on participation in research in Malawi. There is a lack of guidance on appropriate ways to involve children in research in low-income country settings, and the article contributes to the limited evidence base. We found that parents and children largely agree on the importance of involving children in decisions on research, and about the need to promote children’s decision-making capacity while respecting parents’ interests in children’s welfare. Their views also highlight the need to consider specific contexts such as child-headed households, and the importance of flexible approaches that suit different children’s capacities, family environments and study types. Further discussion within the Malawi research and ethics community will help develop contextually-appropriate assent guidelines.

## Abbreviations

COMREC: College of Medicine Research Ethics Committee
FGD: Focus Group discussion
LMIC: Low and medium income countries
MLW: Malawi-Liverpool Wellcome Trust Clinical Research Programme
